# Comprehensive Analysis of Key Genes and Regulatory Elements in Osteosarcoma Affected by Bone Matrix Mineral With Prognostic Values

**DOI:** 10.3389/fgene.2020.00533

**Published:** 2020-06-03

**Authors:** Mi Li, Xin Jin, Hao Li, Caihong Yang, Sisi Deng, Gang Wu

**Affiliations:** ^1^Department of Orthopedics, Tongji Hospital, Tongji Medical College, Huazhong University of Science and Technology, Wuhan, China; ^2^Department of Digestive Surgical Oncology, Union Hospital, Tongji Medical College, Huazhong University of Science and Technology, Wuhan, China; ^3^Cancer Center, Union Hospital, Tongji Medical College, Huazhong University of Science and Technology, Wuhan, China

**Keywords:** osteosarcoma, matrix mineral, protein-protein interaction network, differentially expressed genes, regulatory elements

## Abstract

Osteosarcoma is one of the most common types of bone sarcoma with a poor prognosis. However, genes involved in the mineral metabolism in the microenvironment of the bone affected by osteosarcoma are, to date, largely unknown. A public data series (GSE114237) was used to identify differentially expressed genes (DEGs) between osteosarcoma cells adhering to demineralized osseous surfaces and mineralized osseous surfaces. Functional enrichment analysis of DEGs and hub genes, protein-protein interaction network of DEGs and regulatory network (miRNA-mRNA network and transcription factor (TF)-mRNA network), survival analysis of hub genes was visualized. The prognostic hub genes were considered as candidate genes and their functional predictions were analyzed. A total of 207 DEGs were mainly enriched in extracellular space and thirteen hub genes were mainly enriched in the function of epithelial to mesenchymal transition. However, out of these, only one candidate gene was found to be suitable as a candidate gene. Besides that, 297 miRNAs and 349 TFs interacting with the hub genes were screened. In conclusion, the DEGs, hub genes, miRNAs and TFs screened out in this research could contribute to comprehend the latent mechanisms in osteosarcoma affected by matrix mineral and provide potential research molecular for further study.

## Introduction

Osteosarcoma is one of the most common types of bone sarcoma, accounting for approximately 20% of newly diagnosed bone cancers (Klein and Siegal, 2006; Siegel et al., 2018). The prevalence of osteosarcoma is related to bone growth and is the most malignant tumors in adolescents (Mirabello et al., 2009). Unlike the other tissues, there is a lot of mineral in the microenvironment matrix of bone tissue, which may play a potential role in the tumorigenesis of osteosarcoma. When mesenchymal stem cells (MSCs) were embedded in a calcified ceramic scaffold, tumoral bone formation was observed in the surroundings scaffold (Rubio et al., 2014). *In vitro* experiments showed that CA^2+^ supplementation can provide bone mineralization (Flammini et al., 2016). Meanwhile, selenium-doped hydroxyapatite nanoparticles could induce apoptosis of tumor cells by an inherent caspase-dependent apoptosis pathway together with the generation of reactive oxygen(Wang et al., 2016). Osteosarcoma cell lines have been reported can act as osteoblasts when grown on calcified materials (Zhang et al., 2013). However, the mechanisms of the mineral in the microenvironment affected by osteosarcoma are, to date, still largely unknown.

Bioinformatics, as a new technology, has been widely developed in recent years and plays an important role in revealing the internal mechanism of tumor progression and carcinogenesis at the genome level. Till date, numerous studies have been published on different cancer types, and some researches have revealed the interesting information of the expression of key genes, microRNAs and co-expression modules in osteosarcoma(Li et al., 2019; Li et al., 2020) and drug resistance in osteosarcoma patients(Bhuvaneshwar et al., 2019).

Here we analyzed the microarray dataset obtained from osteosarcoma cells adhered to the demineralized osseous surfaces and mineralized osseous surfaces from the Gene Expression Omnibus (GEO) database to identify differentially expressed genes (DEGs). Subsequently, a protein-protein interaction (PPI) network was constructed to screen out hub genes in the DEGs. Gene Ontology (GO) and Kyoto Encyclopedia of Genes and Genomes (KEGG) pathway enrichment analyses of the DEGs and the hub genes were used to reveal the underlying mechanisms. Subsequently, we constructed regulatory networks of the hub genes. Furthermore, the hub genes survival analysis was carried out to find some candidate genes and to predict the possible function of these genes. Taken together, a total of 207 DEGs, 297 miRNAs, 349 transcription factors, 13 hub genes, and 1 candidate gene were identified.

## Materials and Methods

### Identification of DEGs and Construction of PPI Network

A public series submitted by Wischmann et al. in 2018, GSE114237 (Wischmann et al., 2018), was downloaded from the GEO^1^ (Barrett et al., 2013). The researchers had prepared 8 groups of porcine tibia slices; four groups were demineralized whereas the others were left in their native condition. Later, osteosarcoma cells (MG-63 cells) were seeded onto the osseous surfaces for 72 h. All the expression data were analyzed using the R language (version 3.5.1) BIOCONDUCTOR package, and the DEGs were screened out via the LIMMA package. The cutoff of statistical significance was *p*-values < 0.05 and an absolute value of fold change of gene expression greater than two. The STRING database (online Search Tool for the Retrieval of Interacting Genes,^2^) (Szklarczyk et al., 2017) was used to predict and construct PPI network with a statistical significance of interaction scores of >0.4 (medium confidence score), and the network was visualized by Cytoscape software (version 3.7.0, RRID: SCR_003032; the National Resource for Network Biology [NRNB], Bethesda, MD, United States) (Smoot et al., 2011). Subsequently, hub genes were selected via MCODE (Molecular Complex Detection, RRID: SCR_015828) (Bandettini et al., 2012), the app of Cytoscape, employing the following criteria: MCODE score >5.5, degree cut-off = 2, score cut-off = 0.2, max depth = 100, and k-score = 2.

### Functional and Pathway Enrichment

DAVID^3^ (Huang et al., 2009) was used to perform the biological process (BP), cellular component (CC), molecular function (MF) analysis (Gene Ontology Consortium, 2008), and KEGG (Kanehisa and Goto, 2000) pathway enrichment analysis. The DEGs and hub genes were carried out separately with an indicated statistical significance of *p*-value < 0.05.

### Regulatory Network of the Hub Genes

The miRDB^4^ (Wong and Wang, 2015) online database was used to determine the potential miRNAs that may bind to the hub genes, which were filtered by a confidence score >60. Additionally, the AnimalTFDB 3.0^5^ (Hu et al., 2019), was used to further determine the potential transcription factors (TF) that might interact with hub genes and regulate their expression, filtered with *Q*-values < 0.01. The miRNA-mRNA and TF-mRNA network were visualized via Cytoscape (Smoot et al., 2011).

### Survival Analysis of the Hub Genes

All the hub genes were input into the PROGgeneV2^6^ database (Goswami and Nakshatri, 2014) separately, and the overall survival plot (Kaplan Meier, KM plot) for each gene was created. According to the median gene expression, the data were divided into high and low expression groups. PROGgeneV2 evaluates the prognostic implications of genes using the SURVIVAL package of the R language for a hypothesis test via publicly available data. The hub genes were considered as candidate genes with a *p*-value less than 0.05 for further analysis.

### Prediction of Functions of the Candidate Genes

The candidate genes were submitted to the GeneMANIA^7^ (Warde-Farley et al., 2010) to illustrate the interactive functional association network among the candidate genes and their co-expression genes. The advanced statistical options used were as follows: max resultant genes = 20, max resultant attributes = 10, using the automatically selected weighing method. Subsequently, the Anatomic Viewer of the online SAGE database (Serial Analysis of Gene Expression database,^8^) (Boon et al., 2002) displayed the expression of candidate genes in normal and corresponding malignant human tissues. The related expression levels were ordered by different colors based on the counts of SAGE tags. Furthermore, the Open Targets Platform^9^ (Carvalho-Silva et al., 2019) was used to depict relationships between the candidate genes as drug targets and diseases involved in neoplasm and skeletal system disease.

The parameter selection of all these tools above is based on the developers’ references to select the most appropriate parameters.

## Results

### Identification of DEGs and Construction of PPI Network

**FIGURE 1:**
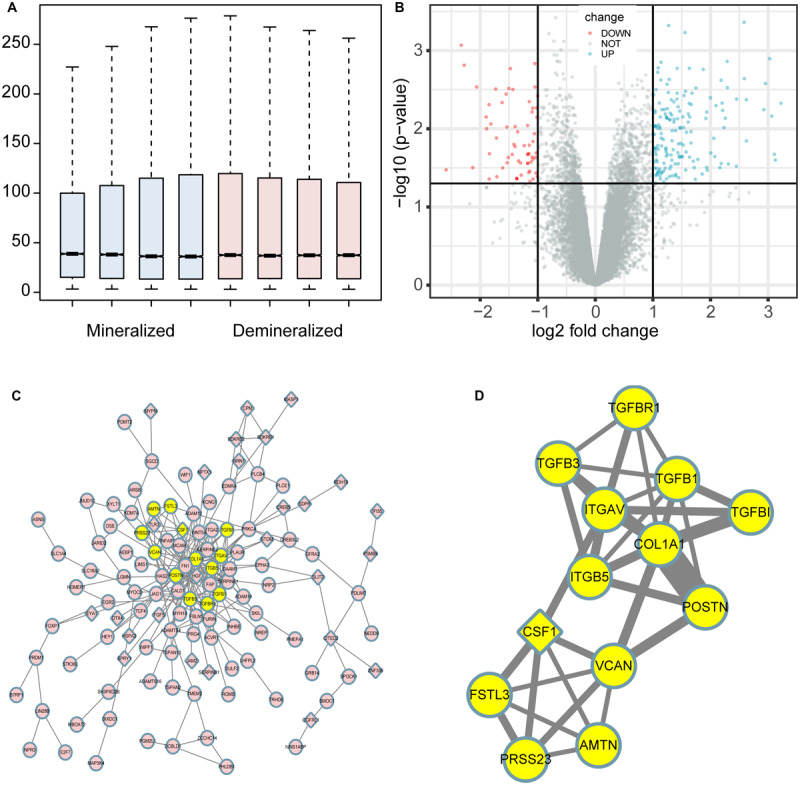
Boxplot, volcano plot, protein-protein interaction (PPI) network, and the most significant module of the DEGs are shown. The boxplot demonstrated that the profiles of GSE114237 had a good consistency **(A)**. The volcano plot exhibited the expression of genes between mineralized and demineralized groups with a cut-off criterion of *p*-value < 0.01 and absolute value logFC (fold change) >1; the red dots represent downregulated genes, the green dots represent upregulated genes, and the gray dots represent unchanged genes **(B)**. The PPI network of the DEGs was constructed and visualized by Cytoscape **(C)**; the round nodes represent upregulated proteins, the diamond nodes represent downregulated proteins, and the edges represent protein-protein associations. Besides, the yellow nodes represent the most significant module obtained from the PPI network **(D)**; the width of the edges represents the coexpression value between the nodes.

The profiles of the public series from GEO had a good consistency shown in Figure 1A. In total, 207 DEGs were identified in the demineralized group; approximately 151 upregulated and 56 downregulated genes were identified (Figure 1B). As we know, the *p*-value calculation includes the number of genes we input for the enrichment and the total number of genes set in a specific concentration; it means that if we analyze the up-and down-regulated genes together, we will get a smaller and more significant *p*-value. In this way, some underestimating important significant pathways can be avoided. The PPI network constructed via Cytoscape of the DEGs as shown in Figure 1C, while the most significant module in Figure 1D with 13 nodes and 33 edges. These 13 genes were proposed as hub genes (12 upregulated genes and 1 downregulated gene). The detailed information is provided in Table 1. The network of hub genes may be an important regulatory mechanism in osteosarcoma affected by matrix mineral.

**TABLE 1:** The statistical metrics for the hub genes.

**ID**	**Gene symbol**	***p* value**	***t* value**	**Log FC**	**Gene title**
8095508	AMTN	0.025101	2.723250	3.127518	amelotin
8016646	COL1A1	0.030041	2.609746	1.312103	collagen type I alpha 1 chain
7903786	CSF1	0.016229	−3.000870	−1.147710	colony stimulating factor 1
8023995	FSTL3	0.001664	4.555960	1.091519	follistatin like 3
8046861	ITGAV	0.019769	2.874816	1.229955	integrin subunit alpha V
8090162	ITGB5	0.027612	2.662971	1.191944	integrin subunit beta 5
7971077	POSTN	0.006454	3.604433	1.214450	periostin
7942957	PRSS23	0.032420	2.561707	1.294606	protease, serine 23
8037005	TGFB1	0.007334	3.519042	1.241910	transforming growth factor beta 1
7980316	TGFB3	0.005699	3.688186	2.927218	transforming growth factor beta 3
8108217	TGFBI	0.007061	3.544310	1.128630	transforming growth factor beta induced
8156826	TGFBR1	0.005879	3.667198	1.037752	transforming growth factor beta receptor 1
8106743	VCAN	0.009674	3.336135	2.234149	versican

### Functional and Pathway Enrichment

**FIGURE 2:**
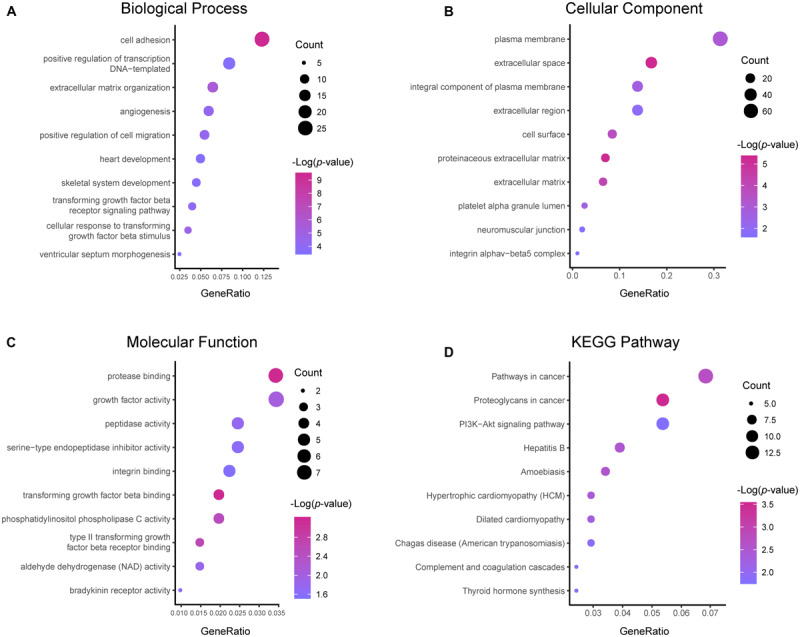
Functional and pathway enrichment analyses of the DEGs. These include the biological process **(A)**, cellular component **(B)**, molecular function **(C)** and KEGG pathway **(D)**; the size of the dots represents the gene count and the color depth of the dots represents the -log (*p*-value).

**FIGURE 3:**
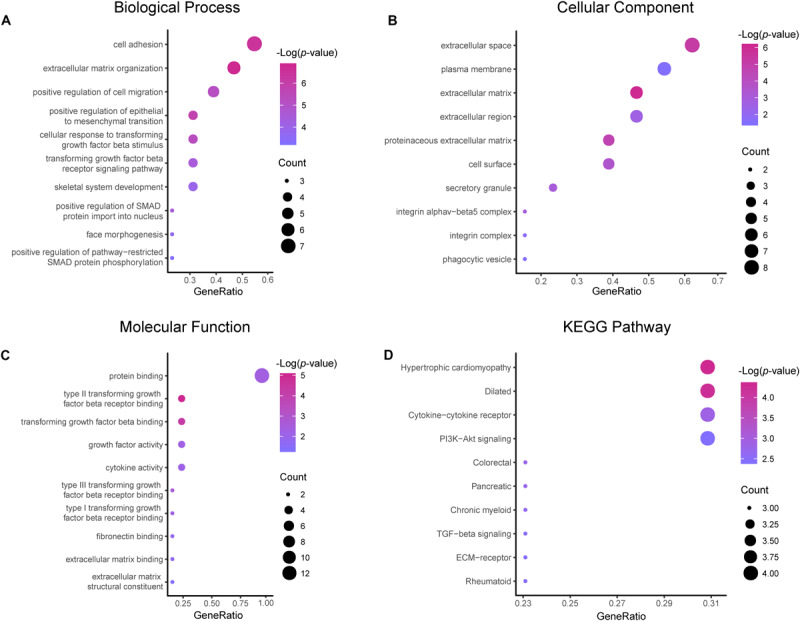
Functional and pathway enrichment analyses of the hub genes. These include the biological process **(A)**, cellular component **(B)**, molecular function **(C)** and KEGG pathway **(D)**; the size of the dots represents the gene count and the color depth of the dots represents the -log (*p*-value).

Results of the GO and KEGG pathway enrichment analyses of the DEGs are shown in Figure 2 (details are shown in Tables 2, 3). In the BP ontology (Figure 2A), cell adhesion (25 genes) and extracellular matrix organization (13 genes) were the most significantly enriched terms. In the CC ontology (Figure 2B), the terms were related to extracellular space (34 genes) and plasma membrane (64 genes). While the terms in the MF ontology (Figure 2C), were related to transforming growth factor-beta binding (4 genes) and protease binding (7 genes). Besides that, in the KEGG pathways (Figure 2D), the terms were revealed to be proteoglycans in cancer (11 genes) and pathways in cancer (14 genes). Particularly, GO and KEGG pathway enrichment analysis of the hub genes showed that they were mainly related to the positive regulation of epithelial to mesenchymal transition, extracellular matrix, type II transforming growth factor-beta receptor binding, and hypertrophic cardiomyopathy (Figure 3, also can be seen in Supplementary Tables 1, 2). These enrichment results reveal that transforming growth factor-beta binding function, especially in the extracellular matrix, should need more attention.

**TABLE 2:** The top five enriched GO terms of the DEGs.

**Category**	**GO ID**	**GO term**	**Count**	**FDR**	**Log P**	**Genes**
Biological process	0007155	cell adhesion	25	6.55E-07	−9.41	PRKCA, NRP2, ATP1B1, AMTN, ITGA2, PCDHB2, NEDD9, ITGB5, SPOCK1, POSTN, MCAM, EPHA3, TNFAIP6, RGMB, KIAA1462, ITGAV, FAP, CDON, TGFBI, VCAN, COL1A1, ADAM12, BOC, FN1, MYH10
Biological process	0030198	extracellular matrix organization	13	0.00382	−5.64	OLFML2B, ITGA2, ITGB5, POSTN, FURIN, ITGAV, FBLN5, SERPINE1, TGFBI, VCAN, COL1A1, JAM2, FN1
Biological process	0071560	cellular response to transforming growth factor beta stimulus	7	0.029311	−4.76	WNT5A, LIMS1, TGFBR1, POSTN, PDE3A, COL1A1, TGFB1
Biological process	0030335	positive regulation of cell migration	11	0.075174	−4.35	PRKCA, TNFAIP6, ITGAV, CSF1, TGFBR1, HAS2, COL1A1, HGF, MCAM, TGFB1, ACVR1
Biological process	0001525	angiogenesis	12	0.076519	−4.34	NRP2, PRKCA, FMNL3, HEY1, ITGAV, FAP, PLXDC1, TGFBI, SERPINE1, JAG1, MCAM, FN1
Cellular component	0005615	extracellular space	34	0.006268	−5.31	WNT5A, FGF5, AEBP1, CPM, MASP1, CSF1, FSTL3, TGFB3, SPOCK1, POSTN, NRN1, TGFB1, ALDH3A1, SERPINE2, IFNE, FAP, TGFBI, SERPINE1, FN1, HGF, MCAM, FURIN, SLIT2, STOM, TNFAIP6, SULF2, INHBE, FBLN5, PLXDC1, FABP3, SERPINB1, VCAN, COL1A1, ADAMTS4
Cellular component	0005578	proteinaceous extracellular matrix	14	0.007391	−5.23	WNT5A, AMTN, ADAMTS16, OLFML2B, POSTN, SPOCK1, TGFB1, SLIT2, SMOC1, FBLN5, TGFBI, VCAN, ADAMTS4, FN1
Cellular component	0031012	extracellular matrix	13	0.098728	−4.11	AEBP1, TGFB3, POSTN, TGFB1, SERPINE2, FBLN5, CDON, SERPINE1, TGFBI, VCAN, COL1A1, FN1, ADAMTS4
Cellular component	0009986	cell surface	17	0.279007	−3.66	WNT5A, ARSB, CPM, TGFBR1, TGFB3, ITGB5, TLR3, ITGA2, ADGRG2, FURIN, SLIT2, TGFB1, SLC1A4, HSPA2, SULF2, ITGAV, FAP
Cellular component	0005886	plasma membrane	64	1.063098	−3.07	SLC5A3, NRP2, ATP1B1, SLC44A1, SCN3A, TGFB3, JAG1, SLC26A2, TGFB1, EDNRA, SLC1A4, SPRY1, FAP, TGFBI, SERPINE1, NALCN, SLC4A4, BOC, KCNG1, PRKCA, FMNL3, PCDHB2, SLIT2, PLAUR, AMIGO2, PLCE1, PLXDC1, SGCD, JAM2, ADAM12, STEAP2, ARL4C, GRB14, ICOSLG, PMEPA1, WNT5A, ABCA8, LIMS1, CPM, CALD1, CSF1, ITGB5, BDKRB1, BDKRB2, NRN1, DAAM1, ALDH3A1, SERINC5, RGMB, ITGAV, TGFBR1, TSPAN13, ITGA2, NPR3, MCAM, FURIN, EPHA3, DOCK4, SULF2, CDON, SYTL5, RGS9, GFRA2, MYH10
Molecular function	0050431	transforming growth factor beta binding	4	0.914081	−3.19	ITGAV, TGFBR1, TGFB3, ACVR1
Molecular function	0002020	protease binding	7	1.19624	−3.07	ITGAV, FAP, SERPINE1, BDKRB2, FURIN, FN1, ADAMTS4
Molecular function	0005114	type II transforming growth factor beta receptor binding	3	3.348849	−2.62	TGFBR1, TGFB3, TGFB1
Molecular function	0004435	phosphatidylinositol phospholipase C activity	4	4.30035	−2.51	EDNRA, PLCE1, PLCB4, BDKRB2
Molecular function	0008083	growth factor activity	7	11.86587	−2.05	FGF5, INHBE, CSF1, TGFB3, JAG1, HGF, TGFB1

**TABLE 3:** The enriched KEGG pathway terms of the DEGs.

**Pathway ID**	**Pathway name**	**Gene count**	**FDR**	**LogP**	**Genes**
hsa05205	Proteoglycans in cancer	11	0.384572	−3.51	PRKCA, WNT5A, PLCE1, ITGAV, ITGB5, ITGA2, HGF, MIR21, TGFB1, FN1, PLAUR
hsa05200	Pathways in cancer	14	2.496893	−2.69	PRKCA, WNT5A, FGF5, TGFBR1, TGFB3, ITGA2, BDKRB1, HGF, BDKRB2, TGFB1, EDNRA, PLCB4, ITGAV, FN1
hsa05146	Amoebiasis	7	3.449882	−2.55	PRKCA, PLCB4, TGFB3, SERPINB1, COL1A1, TGFB1, FN1
hsa05161	Hepatitis B	8	3.819504	−2.50	PRKCA, EGR2, TGFBR1, TGFB3, CREB3L2, TLR3, CREB5, TGFB1
hsa05410	Hypertrophic cardiomyopathy (HCM)	6	4.545801	−2.43	ITGAV, TGFB3, SGCD, ITGB5, ITGA2, TGFB1
hsa05414	Dilated cardiomyopathy	6	6.194945	−2.29	ITGAV, TGFB3, SGCD, ITGB5, ITGA2, TGFB1
hsa05142	Chagas disease (American trypanosomiasis)	6	14.38482	−1.90	PLCB4, TGFBR1, SERPINE1, TGFB3, BDKRB2, TGFB1
hsa04610	Complement and coagulation cascades	5	15.32817	−1.87	MASP1, SERPINE1, BDKRB1, BDKRB2, PLAUR
hsa04918	Thyroid hormone synthesis	5	16.03452	−1.85	PRKCA, ATP1B1, PLCB4, CREB3L2, CREB5
hsa04151	PI3K−Akt signaling pathway	11	18.46895	−1.79	PRKCA, FGF5, ITGAV, CSF1, CREB3L2, ITGB5, ITGA2, CREB5, COL1A1, HGF, FN1
hsa04919	Thyroid hormone signaling pathway	6	20.70627	−1.73	PRKCA, SLC16A2, ATP1B1, PLCE1, PLCB4, ITGAV
hsa04022	cGMP-PKG signaling pathway	7	20.92307	−1.73	EDNRA, ATP1B1, PLCB4, CREB3L2, CREB5, PDE3A, BDKRB2
hsa04961	Endocrine and other factor-regulated calcium reabsorption	4	23.96458	−1.66	PRKCA, ATP1B1, PLCB4, BDKRB2
hsa04350	TGF-beta signaling pathway	5	27.4941	−1.59	INHBE, TGFBR1, TGFB3, TGFB1, ACVR1
hsa04911	Insulin secretion	5	28.40853	−1.57	PRKCA, ATP1B1, PLCB4, CREB3L2, CREB5
hsa04512	ECM-receptor interaction	5	30.26743	−1.54	ITGAV, ITGB5, ITGA2, COL1A1, FN1
					

### Regulatory Network of the Hub Genes

**FIGURE 4:**
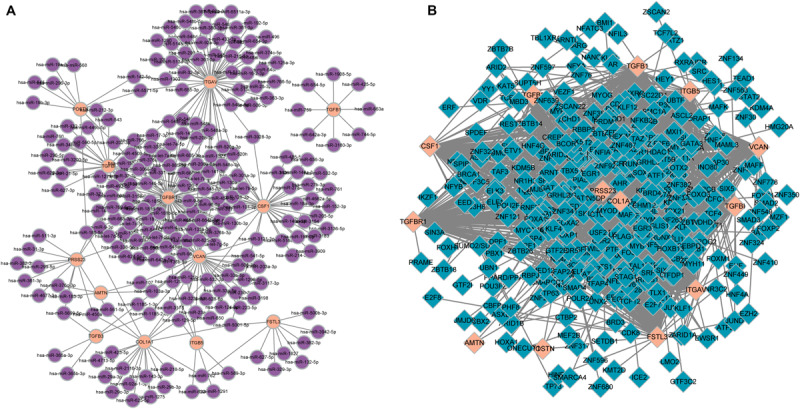
miRNA-mRNA **(A)** and transcription factor-mRNA **(B)** regulatory networks of the hub genes; the orange nodes represent hub genes, the purple nodes represent miRNAs, and the dark blue nodes represent transcription factors.

In total, 297 miRNAs and 349 TFs were screened out employing a relative database (details in Methods). The miRNA-mRNA (Figure 4A) and TF-mRNA (Figure 4B) interaction regulatory networks were constructed and visualized with that the orange nodes represent hub genes, the purple nodes miRNAs, and the dark blue nodes TFs by Cytoscape. The top 10 miRNAs that regulated the hub genes, ranked by topological coefficients, were hsa-miR-664b-3p, hsa-miR-135a-5p, hsa-miR-548an, hsa-miR-135b-5p, hsa-miR-301a-3p, hsa-miR-301b-3p, hsa-miR-454-3p, hsa-miR-130b-3p, hsa-miR-130a-3p, and hsa-miR-128-3p. Furthermore, the top 10 TFs interacted with the hub genes, ranked by topological coefficients, were VEZF1, CHD1, POLR2A, TAF1, RUNX1T1, HCFC1, ELK4, ZEB1, CDK8, and ZNF22. The topological properties of the miRNAs and TFs are displayed in Tables 4, 5. The miRNA-mRNA network and TF-mRNA network predict the potential mechanism, which can provide evidence for subsequent experimental verification.

**TABLE 4:** The top 10 miRNAs that regulated the hub genes and their topological properties.

**Name**	**Average shortest path length**	**Betweenness centrality**	**Closeness centrality**	**Degree**	**Neighborhood connectivity**	**Radiality**	**Stress**	**Topological coefficient**
hsa-miR-664b-3p	3.12931	0.007646	0.319559	2	56.5	0.733836	8180	0.549505
hsa-miR-135a-5p	3.12931	0.007646	0.319559	2	56.5	0.733836	8180	0.549505
hsa-miR-548an	3.12931	0.007646	0.319559	2	56.5	0.733836	8180	0.549505
hsa-miR-135b-5p	3.12931	0.007646	0.319559	2	56.5	0.733836	8180	0.549505
hsa-miR-301a-3p	3.094828	0.009452	0.32312	2	56.5	0.738147	8298	0.544118
hsa-miR-301b-3p	3.094828	0.009452	0.32312	2	56.5	0.738147	8298	0.544118
hsa-miR-454-3p	3.094828	0.009452	0.32312	2	56.5	0.738147	8298	0.544118
hsa-miR-130b-3p	3.094828	0.009452	0.32312	2	56.5	0.738147	8298	0.544118
hsa-miR-130a-3p	3.094828	0.009452	0.32312	2	56.5	0.738147	8298	0.544118
hsa-miR-128-3p	3.094828	0.009452	0.32312	2	56.5	0.738147	8298	0.544118
								

**TABLE 5:** The top 10 transcription factors that regulated the hub genes and their topological properties.

**Name**	**Average shortest path length**	**Betweenness centrality**	**Closeness centrality**	**Degree**	**Neighborhood connectivity**	**Radiality**	**Stress**	**Topological coefficient**
VEZF1	2.628809	4.56E-05	0.3804	2	195.5	0.592798	2458	0.813808
CHD1	2.628809	4.56E-05	0.3804	2	195.5	0.592798	2458	0.813808
POLR2A	2.628809	4.56E-05	0.3804	2	195.5	0.592798	2458	0.813808
TAF1	2.628809	4.56E-05	0.3804	2	195.5	0.592798	2458	0.813808
RUNX1T1	2.628809	4.56E-05	0.3804	2	195.5	0.592798	2458	0.813808
HCFC1	2.628809	4.56E-05	0.3804	2	195.5	0.592798	2458	0.813808
ELK4	2.512465	7.01E-05	0.398015	2	203.5	0.621884	4016	0.778846
ZEB1	2.512465	7.01E-05	0.398015	2	203.5	0.621884	4016	0.778846
CDK8	2.512465	7.01E-05	0.398015	2	203.5	0.621884	4016	0.778846
ZNF22	2.67313	4.70E-05	0.374093	2	180	0.581717	2676	0.774892

### Survival Analysis of the Hub Genes

**FIGURE 5:**
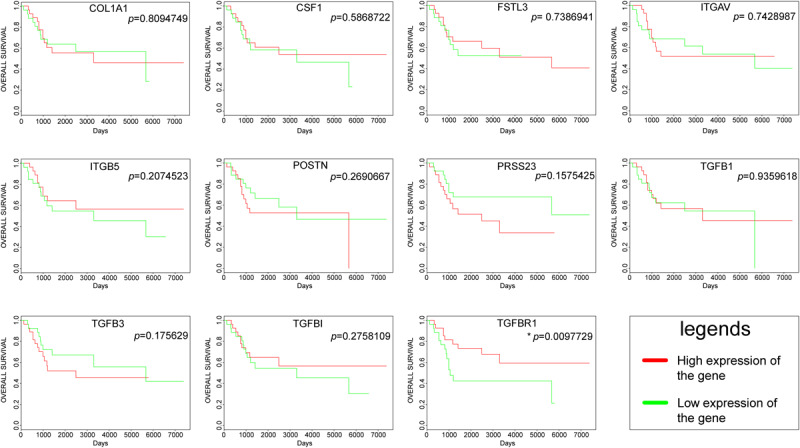
Survival curves of hub genes were created by the PROGgeneV2 online platform; the red line represents a high expression of the genes, and the green line represents a low expression of the genes.

The overall survival plots could be obtained for only 11 hub genes out of 13 hub genes, as shown in Figure 5. However, most of them were not statistically significant. Unfortunately, there was no significant difference in the survival curve of COL1A1, CSF1, FSTL3, ITGAV, ITGB5, POSTN, PRSS23, TGFB1, TGFB3, and TGFBI, and no data result for AMTN and VCAN. Only one gene, namely TGFBR1, had a *p*-value < 0.05. High expression of TGFBR1 had better overall survival.

Since TGFBR1 is one of the hub genes involved in the regulation mechanism in osteosarcoma affected by matrix mineral, and its differential expression will affect the prognosis and survival of patients. Therefore, TGFBR1 was considered as a candidate gene for further analyses.

### Prediction of Functions of the Candidate Genes

**FIGURE 6:**
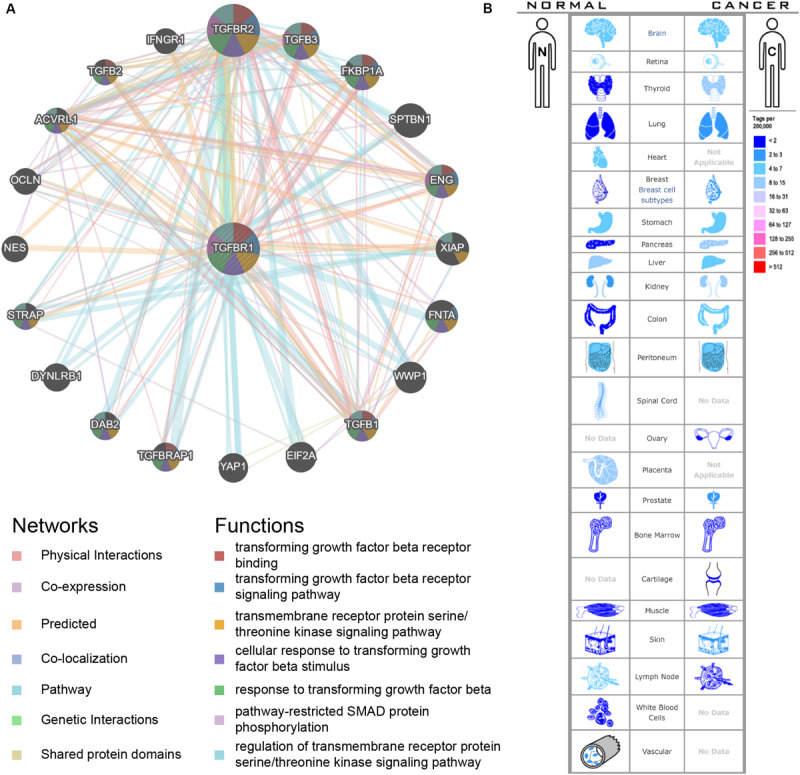
The functional association network **(A)** and expression profiles **(B)** of TGFBR1. In the functional association network, the edge-colors represent the bioinformatics method applied; the node-colors represent the biological functions of the set of enrichment genes. In the expression profiles, the left side represents normal tissues and the right side represents the matched cancer tissues. The related expression levels are based on analysis of the counts of SAGE tags, ordered by 10 colors.

**FIGURE 7:**
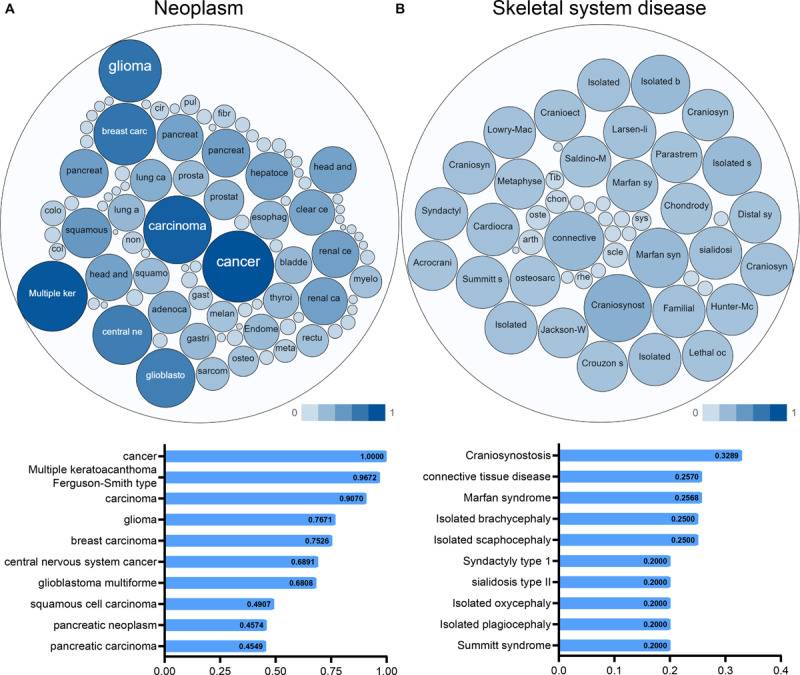
The target-disease association related to TGFBR1 in neoplasm **(A)** and skeletal system disease **(B)** could be determined and viewed by the Open Targets Platform; the bubbles color and bubbles size represent the association score, and the top 10 diseases with a high score value are listed below.

An interactive functional association network constructed by GeneMANIA revealed correlations among genes for the candidate gene. The gene set enriched for TGFBR1 is responsible mainly for the pathways related to TGFB (transforming growth factor-beta) receptor binding and TGFB receptor signaling (Figure 6A). In our study, the expression profiles of the TGFBR1 in human tissue are displayed using SAGE. As shown, TGFBR1 mRNA in thyroid, lung, pancreas, colon, and prostate cancer tissues displayed higher levels than in the matched normal tissues (Figure 6B). Subsequently, the target-disease association related to TGFBR1 in neoplasm and skeletal system disease could be determined and viewed by using the Open Targets Platform. Among the diseases, the top five diseases with a high score of TGFBR1-neoplasm association were cancer, multiple keratoacanthomata, Ferguson-Smith type carcinoma, glioma, and breast carcinoma (Figure 7A). Furthermore, the top five diseases with a high score of TGFBR1-skeletal system disease association were craniosynostosis, connective tissue disease, Marfan syndrome, isolated brachycephaly, and isolated scaphocephaly (Figure 7B). All these findings predict the function of TGFBR1 and can be helpful for further research.

## Discussion

The mineralization of the bone matrix plays a potentially important role in the process of bone formation. Osteosarcoma is an intraosseous malignancy that occurs predominantly in growing skeletal regions (Alfranca et al., 2015). The extracellular mineral is the main inorganic component of the bone matrix. Zhang et al. reported that osteosarcoma cell lines altered the expression of cytokines when grown on calcified materials (Zhang et al., 2013). Meanwhile, Kingsley et al. found that calcium is released into bone microenvironment through osteoclastic bone resorption, leading to a “vicious circle” by its participation in different mechanisms of Parathyroid Hormone Related Protein (PTHRP) production (Kingsley et al., 2007). Meanwhile, Rubio et al. have demonstrated the role of microenvironment calcium matrix in the development of the osteosarcoma via embedding osteosarcoma tumor cells in calcium phosphate biomaterials (Rubio et al., 2014).

In our study, a microarray dataset was analyzed to obtain DEGs from osteosarcoma cells adhering to the demineralized osseous surfaces against mineralized osseous surfaces. Altogether, 207 DEGs comprised 151 upregulated and 56 downregulated genes were identified. Subsequently, enrichment analyses of the DEGs were used to explore some other interactions. Previous studies have reported that extracellular space in the bone microenvironment is essential for osteosarcoma development (Cai et al., 2015; Baglio et al., 2017; Cortini et al., 2017; Zheng et al., 2018). Moreover, studies have revealed that cell adhesion is a significant process of osteosarcoma, which involves a number of molecules (Gang et al., 2017; Hu et al., 2017; Villanueva et al., 2019). In other words, all these studies are consistent with the results obtained in our studies. In contrast, the functional analysis demonstrated the hub genes were mainly related to the positive regulation of epithelial to mesenchymal transition, extracellular matrix, type II transforming growth factor-beta receptor binding, and hypertrophic cardiomyopathy.

The hub genes were selected in the DEGs PPI network of the most significant module. Further survival analysis revealed TGFBR1 as a candidate gene because of its statistical significance. Meanwhile, some hub genes have been reported involved in the tumorigenesis and progression of osteosarcoma, such as ITGAV, POSTN, COL1A1, TGFB1, TGFB3, and ITGAV. These can be negatively regulated by miR-548c-3p, just like the target agents against integrin as reported in clinical trials, and can be potential tumor therapeutic targets (Luo et al., 2016). Moreover, high expression of POSTN (periostin, also known as osteoblast-specific factor two) in osteosarcoma is significantly associated with angiogenesis and is considered to be a promising prognostic factor in osteosarcoma patients (Hu et al., 2016). Similarly, COL1A1 also has a prognostic value in osteosarcoma patients (He et al., 2014). In osteosarcoma, TGFB signaling could interact with both microenvironment and tumor cells, exerting the characteristic effects of tumor precursors. Therefore, TGFBs could be considered as independent therapeutic targets for osteosarcoma (Lamora et al., 2016; Verrecchia and Redini, 2018; Xie et al., 2018).

In addition, a search of published literature revealed that some hub genes have been associated with tumors. VCAN is reported to be essential for migration and metastasis of breast cancer (Zhang et al., 2019) and could be a potential prognostic biomarker for colon cancer (Chida et al., 2016). Moreover, knockdown of PRSS23 could inhibit EIF2 signaling, thereby suppressing the tumor growth of gastric cancer (Han et al., 2019). Furthermore, one report has demonstrated that CSF1 could promote melanoma resistance to PD1 checkpoint blockade (Neubert et al., 2018), and many reports have shown that CSF1R targeting may be beneficial by showing strong anti-tumor effects (Zhu et al., 2014; Obba et al., 2015; Kumar et al., 2017; Neubert et al., 2018). Similarly, TGFBI has tumor-promoting and tumor-protective roles in certain gastrointestinal tract cancers (Han et al., 2015), and in prostate cancer (Al Shareef et al., 2018).

Particularly, the candidate gene TGFBR1 is reported to be involved in multiple biological functions in tumor. Inhibition of TGFBR1 can block the activation of fibroblasts mediated via IL1B/TGFB1 and reduce the secretion of pro-inflammatory cytokines in colorectal cancer, which would make the cancer cells more sensitive to chemotherapy. Moreover, TGFBR1 inhibitor could reduce the metastasis ability of tumor cells *in vivo* (Guillen Diaz-Maroto et al., 2019). It is also reported that in lung cancer TGFBR1 may be a target of tumor suppressor genes (Gao et al., 2019). However, there are few reports on the function of TGFBR1 in osteosarcoma. Upregulated TGFBR1 will induce the epithelial-to-mesenchymal transition in osteosarcoma cell lines, which is regulated by the interferon consensus sequence-binding protein, but the specific mechanism is not fully elaborated (ICSBP) (Sung et al., 2019).

This bioinformatics study provides information on DEGs and regulatory elements involved in osteosarcoma affected by matrix mineral. However, the mechanism of these molecules are still unknown, and additional well-designed experiments and analyses are required. In addition, all the results from this study were got *in silico*. Further, *in vivo* and *in vitro* experiments are necessary to test the biological functions of the key genes and their regulatory elements.

In conclusion, we identified 207 DEGs, 13 hub genes, 297 miRNAs, 349 TFs, and 1 candidate gene, which may be involved in the processes of matrix mineralization that may provide a functional signal to osteosarcoma cells. The molecules that we found may be potential targets for future research on the microenvironment of osteosarcoma. Furthermore, our results may contribute to the identification of the biomarkers for matrix mineralization related to osteosarcoma.

## Data Availability Statement

The data that support the findings of this study were generated at GSE114237 (Wischmann et al., 2018) in GEO. Derived data supporting the findings of this study are available from the corresponding author on reasonable request.

## Author Contributions

ML and XJ analyzed the data. HL provided the help of the R language. CY suggested online tools. SD and GW designed the project. SD selected the analyzed results and wrote the manuscript. All authors read and approved the final manuscript.

## Conflict of Interest

The authors declare that the research was conducted in the absence of any commercial or financial relationships that could be construed as a potential conflict of interest.

## Footnotes

1 http://www.ncbi.nlm.nih.gov/geo, RRID: SCR_005012

2 http://string-db.org, RRID: SCR_005223

3 https://david.ncifcrf.gov/, RRID: SCR_001881

4 http://mirdb.org/mining.html, RRID: SCR_010848

5 http://www.bioguo.org/AnimalTFDB/, RRID: SCR_001624

6 http://genomics.jefferson.edu/proggene

7 http://www.genemania.org, RRID: SCR_005709

8 http://www.ncbi.nlm.nih.gov/SAGE, RRID: SCR_000796

9 https://www.targetvalidation.org/

## Abbreviations

DEGs: differentially expressed genes
GEO: gene expression omnibus
GO: gene ontology
KEGG: kyoto encyclopedia of genes and genomes
PPI: protein-protein interaction
SAGE: serial analysis of gene expression
STRING: search tool for the retrieval of interacting genes

## References

[B1] Al Shareef Z. Kardooni H. Murillo-Garzon V. Domenici G. Stylianakis E. Steel J. H. (2018). Protective effect of stromal Dickkopf-3 in prostate cancer: opposing roles for TGFBI and ECM-1. *Oncogene* 37 5305–5324. 10.1038/s41388-018-0294-0 29858602 PMC6160402

[B2] Alfranca A. Martinez-Cruzado L. Tornin J. Abarrategi A. Amaral T. De Alava E. (2015). Bone microenvironment signals in osteosarcoma development. *Cell Mol. Life Sci.* 72 3097–3113. 10.1007/s00018-015-1918-y 25935149 PMC11113487

[B3] Baglio S. R. Lagerweij T. Perez-Lanzon M. Ho X. D. Leveille N. Melo S. A. (2017). Blocking tumor-educated MSC paracrine activity halts osteosarcoma progression. *Clin. Cancer Res.* 23 3721–3733. 10.1158/1078-0432.CCR-17-3198 28053020

[B4] Bandettini W. P. Kellman P. Mancini C. Booker O. J. Vasu S. Leung S. W. (2012). MultiContrast delayed enhancement (MCODE) improves detection of subendocardial myocardial infarction by late gadolinium enhancement cardiovascular magnetic resonance: a clinical validation study. *J. Cardiovasc. Magn. Reson.* 14:83. 10.1186/1532-429X-14-83 23199362 PMC3552709

[B5] Barrett T. Wilhite S. E. Ledoux P. Evangelista C. Kim I. F. Tomashevsky M. (2013). NCBI GEO: archive for functional genomics data sets–update. *Nucleic Acids Res.* 41 D991–D995. 10.1093/nar/gks1193 23193258 PMC3531084

[B6] Bhuvaneshwar K. Harris M. Gusev Y. Madhavan S. Iyer R. Vilboux T. (2019). Genome sequencing analysis of blood cells identifies germline haplotypes strongly associated with drug resistance in osteosarcoma patients. *BMC Cancer* 19:357. 10.1186/s12885-019-5474-y 30991985 PMC6466653

[B7] Boon K. Osorio E. C. Greenhut S. F. Schaefer C. F. Shoemaker J. Polyak K. (2002). An anatomy of normal and malignant gene expression. *Proc. Natl. Acad. Sci. U.S.A.* 99 11287–11292. 10.1073/pnas.152324199 12119410 PMC123249

[B8] Cai R. Kawazoe N. Chen G. (2015). Influence of surfaces modified with biomimetic extracellular matrices on adhesion and proliferation of mesenchymal stem cells and osteosarcoma cells. *Colloids Surf. B Biointerfaces* 126 381–386. 10.1016/j.colsurfb.2014.11.050 25516267

[B9] Carvalho-Silva D. Pierleoni A. Pignatelli M. Ong C. Fumis L. Karamanis N. (2019). Open targets platform: new developments and updates two years on. *Nucleic Acids Res.* 47 D1056–D1065. 10.1093/nar/gky1133 30462303 PMC6324073

[B10] Chida S. Okayama H. Noda M. Saito K. Nakajima T. Aoto K. (2016). Stromal VCAN expression as a potential prognostic biomarker for disease recurrence in stage II-III colon cancer. *Carcinogenesis* 37 878–887. 10.1093/carcin/bgw069 27287872

[B11] Cortini M. Avnet S. Baldini N. (2017). Mesenchymal stroma: role in osteosarcoma progression. *Cancer Lett.* 405 90–99. 10.1016/j.canlet.2017.07.024 28774797

[B12] Flammini L. Martuzzi F. Vivo V. Ghirri A. Salomi E. Bignetti E. (2016). Hake fish bone as a calcium source for efficient bone mineralization. *Int. J. Food Sci. Nutr.* 67 265–273. 10.3109/09637486.2016.1150434 26903386

[B13] Gang L. Qun L. Liu W. D. Li Y. S. Xu Y. Z. Yuan D. T. (2017). MicroRNA-34a promotes cell cycle arrest and apoptosis and suppresses cell adhesion by targeting DUSP1 in osteosarcoma. *Am. J. Transl. Res.* 9 5388–5399. 29312491 PMC5752889

[B14] Gao L. Hu Y. Tian Y. Fan Z. Wang K. Li H. (2019). Lung cancer deficient in the tumor suppressor GATA4 is sensitive to TGFBR1 inhibition. *Nat. Commun.* 10:1665. 10.1038/s41467-019-09295-7 30971692 PMC6458308

[B15] Gene Ontology Consortium, (2008). The Gene Ontology project in 2008. *Nucleic Acids Res.* 36 D440–D444. 10.1093/nar/gkm883 17984083 PMC2238979

[B16] Goswami C. P. Nakshatri H. (2014). PROGgeneV2: enhancements on the existing database. *BMC Cancer* 14:970. 10.1186/1471-2407-14-970 25518851 PMC4300843

[B17] Guillen Diaz-Maroto N. Sanz-Pamplona R. Berdiel-Acer M. Cimas F. J. Garcia E. Goncalves-Ribeiro S. (2019). Noncanonical TGFbeta pathway relieves the blockade of IL1beta/TGFbeta-mediated crosstalk between tumor and stroma: TGFBR1 and TAK1 inhibition in colorectal cancer. *Clin. Cancer Res.* 25 4466–4479. 10.1158/1078-0432.CCR-18-3957 30979739

[B18] Han B. Cai H. Chen Y. Hu B. Luo H. Wu Y. (2015). The role of TGFBI (betaig-H3) in gastrointestinal tract tumorigenesis. *Mol. Cancer* 14:64. 10.1186/s12943-015-0335-z 25889002 PMC4435624

[B19] Han B. Yang Y. Chen J. He X. Lv N. Yan R. (2019). PRSS23 knockdown inhibits gastric tumorigenesis through EIF2 signaling. *Pharmacol. Res.* 142 50–57. 10.1016/j.phrs.2019.02.008 30769097

[B20] He M. Wang Z. Zhao J. Chen Y. Wu Y. (2014). COL1A1 polymorphism is associated with risks of osteosarcoma susceptibility and death. *Tumour Biol.* 35 1297–1305. 10.1007/s13277-013-1172-6 24072491

[B21] Hu C. Chen X. Wen J. Gong L. Liu Z. Wang J. (2017). Antitumor effect of focal adhesion kinase inhibitor PF562271 against human osteosarcoma in vitro and in vivo. *Cancer Sci.* 108 1347–1356. 10.1111/cas.13256 28406574 PMC5497929

[B22] Hu F. Shang X. F. Wang W. Jiang W. Fang C. Tan D. (2016). High-level expression of periostin is significantly correlated with tumour angiogenesis and poor prognosis in osteosarcoma. *Int. J. Exp. Pathol.* 97 86–92. 10.1111/iep.12171 27028305 PMC4840243

[B23] Hu H. Miao Y. R. Jia L. H. Yu Q. Y. Zhang Q. Guo A. Y. (2019). AnimalTFDB 3.0: a comprehensive resource for annotation and prediction of animal transcription factors. *Nucleic Acids Res.* 47 D33–D38. 10.1093/nar/gky822 30204897 PMC6323978

[B24] Huang D. W. Sherman B. T. Lempicki R. A. (2009). Systematic and integrative analysis of large gene lists using DAVID bioinformatics resources. *Nat. Protoc.* 4 44–57. 19131956 10.1038/nprot.2008.211

[B25] Kanehisa M. Goto S. (2000). KEGG: kyoto encyclopedia of genes and genomes. *Nucleic Acids Res.* 28 27–30. 10.1038/gene.2015.7 10592173 PMC102409

[B26] Kingsley L. A. Fournier P. G. Chirgwin J. M. Guise T. A. (2007). Molecular biology of bone metastasis. *Mol. Cancer Ther.* 6 2609–2617. 17938257 10.1158/1535-7163.MCT-07-0234

[B27] Klein M. J. Siegal G. P. (2006). Osteosarcoma: anatomic and histologic variants. *Am. J. Clin. Pathol.* 125 555–581. 16627266 10.1309/UC6K-QHLD-9LV2-KENN

[B28] Kumar V. Donthireddy L. Marvel D. Condamine T. Wang F. Lavilla-Alonso S. (2017). Cancer-associated fibroblasts neutralize the anti-tumor effect of CSF1 receptor blockade by inducing PMN-MDSC infiltration of tumors. *Cancer Cell* 32 654.e5–668.e5. 10.1016/j.ccell.2017.10.005 29136508 PMC5827952

[B29] Lamora A. Talbot J. Mullard M. Brounais-Le Royer B. Redini F. Verrecchia F. (2016). TGF-beta signaling in bone remodeling and osteosarcoma progression. *J. Clin. Med.* 5:96. 10.3390/jcm5110096 27827889 PMC5126793

[B30] Li M. Jin X. Guo F. Wu G. Wu L. Deng S. (2019). Integrative analyses of key genes and regulatory elements in fluoride-affected osteosarcoma. *J. Cell. Biochem.* 120 15397–15409. 10.1002/jcb.28807 31037778

[B31] Li M. Jin X. Li H. Wu G. Wang S. Yang C. (2020). Key genes with prognostic values in suppression of osteosarcoma metastasis using comprehensive analysis. *BMC Cancer* 20:65. 10.1186/s12885-020-6542-z 31992246 PMC6988291

[B32] Luo Z. Li D. Luo X. Li L. Gu S. Yu L. (2016). Decreased expression of miR-548c-3p in osteosarcoma contributes to cell proliferation via targeting ITGAV. *Cancer Biother. Radiopharm.* 31 153–158. 10.1089/cbr.2016.1995 27310302

[B33] Mirabello L. Troisi R. J. Savage S. A. (2009). Osteosarcoma incidence and survival rates from 1973 to 2004: data from the surveillance, epidemiology, and end results program. *Cancer* 115 1531–1543. 10.1002/cncr.24121 19197972 PMC2813207

[B34] Neubert N. J. Schmittnaegel M. Bordry N. Nassiri S. Wald N. Martignier C. (2018). T cell-induced CSF1 promotes melanoma resistance to PD1 blockade. *Sci. Transl. Med.* 10:eaan3311. 10.1126/scitranslmed.aan3311 29643229 PMC5957531

[B35] Obba S. Hizir Z. Boyer L. Selimoglu-Buet D. Pfeifer A. Michel G. (2015). The PRKAA1/AMPKalpha1 pathway triggers autophagy during CSF1-induced human monocyte differentiation and is a potential target in CMML. *Autophagy* 11 1114–1129. 10.1080/15548627.2015.1034406 26029847 PMC4590592

[B36] Rubio R. Abarrategi A. Garcia-Castro J. Martinez-Cruzado L. Suarez C. Tornin J. (2014). Bone environment is essential for osteosarcoma development from transformed mesenchymal stem cells. *Stem Cells* 32 1136–1148. 10.1002/stem.1647 24446210

[B37] Siegel R. L. Miller K. D. Jemal A. (2018). Cancer statistics, 2018. *CA Cancer J. Clin.* 68 7–30. 29313949 10.3322/caac.21442

[B38] Smoot M. E. Ono K. Ruscheinski J. Wang P. L. Ideker T. (2011). Cytoscape 2.8: new features for data integration and network visualization. *Bioinformatics* 27 431–432. 10.1093/bioinformatics/btq675 21149340 PMC3031041

[B39] Sung J. Y. Yoon K. Ye S. K. Goh S. H. Park S. Y. Kim J. H. (2019). Upregulation of transforming growth factor-beta type I receptor by interferon consensus sequence-binding protein in osteosarcoma cells. *Biochim. Biophys. Acta Mol. Cell. Res.* 1866 761–772. 10.1016/j.bbamcr.2019.01.015 30710564

[B40] Szklarczyk D. Morris J. H. Cook H. Kuhn M. Wyder S. Simonovic M. (2017). The STRING database in 2017: quality-controlled protein-protein association networks, made broadly accessible. *Nucleic Acids Res.* 45 D362–D368. 10.1093/nar/gkw937 27924014 PMC5210637

[B41] Verrecchia F. Redini F. (2018). Transforming growth factor-beta signaling plays a pivotal role in the interplay between osteosarcoma cells and their microenvironment. *Front. Oncol.* 8:133. 10.3389/fonc.2018.00133 29761075 PMC5937053

[B42] Villanueva F. Araya H. Briceno P. Varela N. Stevenson A. Jerez S. (2019). The cancer-related transcription factor RUNX2 modulates expression and secretion of the matricellular protein osteopontin in osteosarcoma cells to promote adhesion to endothelial pulmonary cells and lung metastasis. *J. Cell Physiol.* 234 13659–13679. 10.1002/jcp.28046 30637720 PMC13483038

[B43] Wang Y. Wang J. Hao H. Cai M. Wang S. Ma J. (2016). *In vitro* and *in vivo* mechanism of bone tumor inhibition by selenium-doped bone mineral nanoparticles. *ACS Nano* 10 9927–9937. 10.1021/acsnano.6b03835 27797178 PMC5198771

[B44] Warde-Farley D. Donaldson S. L. Comes O. Zuberi K. Badrawi R. Chao P. (2010). The GeneMANIA prediction server: biological network integration for gene prioritization and predicting gene function. *Nucleic Acids Res.* 38 W214–W220. 10.1093/nar/gkq537 20576703 PMC2896186

[B45] Wischmann J. Lenze F. Thiel A. Bookbinder S. Querido W. Schmidt O. (2018). Matrix mineralization controls gene expression in osteoblastic cells. *Exp. Cell Res.* 372 25–34. 10.1016/j.yexcr.2018.09.005 30193837 PMC6185740

[B46] Wong N. Wang X. (2015). miRDB: an online resource for microRNA target prediction and functional annotations. *Nucleic Acids Res.* 43 D146–D152. 10.1093/nar/gku1104 25378301 PMC4383922

[B47] Xie L. Yao Z. Zhang Y. Li D. Hu F. Liao Y. (2018). Deep RNA sequencing reveals the dynamic regulation of miRNA, lncRNAs, and mRNAs in osteosarcoma tumorigenesis and pulmonary metastasis. *Cell Death Dis.* 9:772. 10.1038/s41419-018-0813-5 29991755 PMC6039476

[B48] Zhang H. Wu H. Zheng J. Yu P. Xu L. Jiang P. (2013). Transforming growth factor beta1 signal is crucial for dedifferentiation of cancer cells to cancer stem cells in osteosarcoma. *Stem Cells* 31 433–446. 10.1002/stem.1298 23225703

[B49] Zhang Y. Zou X. Qian W. Weng X. Zhang L. Zhang L. (2019). Enhanced PAPSS2/VCAN sulfation axis is essential for Snail-mediated breast cancer cell migration and metastasis. *Cell Death Differ.* 26 565–579. 10.1038/s41418-018-0147-y 29955124 PMC6370781

[B50] Zheng Y. Wang G. Chen R. Hua Y. Cai Z. (2018). Mesenchymal stem cells in the osteosarcoma microenvironment: their biological properties, influence on tumor growth, and therapeutic implications. *Stem Cell Res. Ther.* 9:22. 10.1186/s13287-018-0780-x 29386041 PMC5793392

[B51] Zhu Y. Knolhoff B. L. Meyer M. A. Nywening T. M. West B. L. Luo J. (2014). CSF1/CSF1R blockade reprograms tumor-infiltrating macrophages and improves response to T-cell checkpoint immunotherapy in pancreatic cancer models. *Cancer Res.* 74 5057–5069. 10.1158/0008-5472.CAN-13-3723 25082815 PMC4182950

