# Supplementary material for: Comprehensive Analysis of Key Genes and Regulatory Elements in Osteosarcoma Affected by Bone Matrix Mineral With Prognostic Values
Source: Front Genet. 2020 Jun 3;11:533. doi: 10.3389/fgene.2020.00533 (PMC7283541; doi:10.3389/fgene.2020.00533)
Supplement: Supplementary file 1 [file Table_1.DOCX]

Supplementary table 1 The top 5 enriched GO terms of the hub genes

| Category | GO ID | GO Term | Count | FDR | Log P | Genes |
| --- | --- | --- | --- | --- | --- | --- |
| Biological Process | 0030198 | extracellular matrix organization | 6 | 2.12E-04 | -6.82 | ITGAV, TGFBI, ITGB5, POSTN, VCAN, COL1A1 |
| Biological Process | 0007155 | cell adhesion | 7 | 4.50E-04 | -6.49 | AMTN, ITGAV, TGFBI, ITGB5, POSTN, VCAN, COL1A1 |
| Biological Process | 0010718 | positive regulation of epithelial to mesenchymal transition | 4 | 0.002085 | -5.82 | TGFBR1, TGFB3, COL1A1, TGFB1 |
| Biological Process | 0071560 | cellular response to transforming growth factor beta stimulus | 4 | 0.006995 | -5.30 | TGFBR1, POSTN, COL1A1, TGFB1 |
| Biological Process | 0030335 | positive regulation of cell migration | 5 | 0.008944 | -5.19 | ITGAV, CSF1, TGFBR1, COL1A1, TGFB1 |
| Cellular Component | 0031012 | extracellular matrix | 6 | 7.72E-04 | -6.10 | TGFBI, TGFB3, POSTN, VCAN, COL1A1, TGFB1 |
| Cellular Component | 0005615 | extracellular space | 8 | 0.006613 | -5.17 | CSF1, TGFBI, TGFB3, FSTL3, POSTN, VCAN, COL1A1, TGFB1 |
| Cellular Component | 0005578 | proteinaceous extracellular matrix | 5 | 0.020209 | -4.69 | AMTN, TGFBI, POSTN, VCAN, TGFB1 |
| Cellular Component | 0009986 | cell surface | 5 | 0.309777 | -3.50 | ITGAV, TGFBR1, TGFB3, ITGB5, TGFB1 |
| Cellular Component | 0030141 | secretory granule | 3 | 1.019697 | -2.98 | TGFB3, FSTL3, COL1A1 |
| Molecular Function | 0005114 | type II transforming growth factor beta receptor binding | 3 | 0.009549 | -5.01 | TGFBR1, TGFB3, TGFB1 |
| Molecular Function | 0050431 | transforming growth factor beta binding | 3 | 0.054361 | -4.26 | ITGAV, TGFBR1, TGFB3 |
| Molecular Function | 0034714 | type III transforming growth factor beta receptor binding | 2 | 2.759216 | -2.55 | TGFB3, TGFB1 |
| Molecular Function | 0005515 | protein binding | 12 | 4.571768 | -2.32 | AMTN, ITGAV, CSF1, TGFBR1, TGFBI, TGFB3, FSTL3, ITGB5, POSTN, VCAN, COL1A1, TGFB1 |
| Molecular Function | 0008083 | growth factor activity | 3 | 5.440397 | -2.25 | CSF1, TGFB3, TGFB1 |
