# Supplementary material for: Comprehensive Analysis of Key Genes and Regulatory Elements in Osteosarcoma Affected by Bone Matrix Mineral With Prognostic Values
Source: Front Genet. 2020 Jun 3;11:533. doi: 10.3389/fgene.2020.00533 (PMC7283541; doi:10.3389/fgene.2020.00533)
Supplement: Supplementary file 2 [file Table_2.DOCX]

Supplementary table 2 The enriched KEGG pathway terms of the hub genes

| Pathway ID | Pathway Name | Gene Count | FDR | LogP | Genes |
| --- | --- | --- | --- | --- | --- |
| hsa05410 | Hypertrophic cardiomyopathy (HCM) | 4 | 0.044444 | -4.32 | ITGAV, TGFB3, ITGB5, TGFB1 |
| hsa05414 | Dilated cardiomyopathy | 4 | 0.055516 | -4.23 | ITGAV, TGFB3, ITGB5, TGFB1 |
| hsa04060 | Cytokine-cytokine receptor interaction | 4 | 1.275698 | -2.86 | CSF1, TGFBR1, TGFB3, TGFB1 |
| hsa05210 | Colorectal cancer | 3 | 1.51504 | -2.79 | TGFBR1, TGFB3, TGFB1 |
| hsa05212 | Pancreatic cancer | 3 | 1.662921 | -2.75 | TGFBR1, TGFB3, TGFB1 |
| hsa05220 | Chronic myeloid leukemia | 3 | 2.033114 | -2.66 | TGFBR1, TGFB3, TGFB1 |
| hsa04350 | TGF-beta signaling pathway | 3 | 2.74776 | -2.53 | TGFBR1, TGFB3, TGFB1 |
| hsa04512 | ECM-receptor interaction | 3 | 2.941844 | -2.50 | ITGAV, ITGB5, COL1A1 |
| hsa05323 | Rheumatoid arthritis | 3 | 3.007884 | -2.49 | CSF1, TGFB3, TGFB1 |
| hsa04151 | PI3K-Akt signaling pathway | 4 | 3.46594 | -2.42 | ITGAV, CSF1, ITGB5, COL1A1 |
| hsa05142 | Chagas disease (American trypanosomiasis) | 3 | 4.153901 | -2.34 | TGFBR1, TGFB3, TGFB1 |
| hsa05146 | Amoebiasis | 3 | 4.308704 | -2.33 | TGFB3, COL1A1, TGFB1 |
| hsa05200 | Pathways in cancer | 4 | 4.983831 | -2.26 | ITGAV, TGFBR1, TGFB3, TGFB1 |
| hsa04380 | Osteoclast differentiation | 3 | 6.448114 | -2.15 | CSF1, TGFBR1, TGFB1 |
| hsa04068 | FoxO signaling pathway | 3 | 6.729188 | -2.13 | TGFBR1, TGFB3, TGFB1 |
| hsa05161 | Hepatitis B | 3 | 7.801778 | -2.06 | TGFBR1, TGFB3, TGFB1 |
| hsa04390 | Hippo signaling pathway | 3 | 8.413716 | -2.03 | TGFBR1, TGFB3, TGFB1 |
| hsa05205 | Proteoglycans in cancer | 3 | 14.04029 | -1.79 | ITGAV, ITGB5, TGFB1 |
| hsa04510 | Focal adhesion | 3 | 14.79698 | -1.77 | ITGAV, ITGB5, COL1A1 |
| hsa04010 | MAPK signaling pathway | 3 | 21.11874 | -1.60 | TGFBR1, TGFB3, TGFB1 |
| hsa05166 | HTLV-I infection | 3 | 21.25967 | -1.60 | TGFBR1, TGFB3, TGFB1 |
| hsa05144 | Malaria | 2 | 37.3978 | -1.31 | TGFB3, TGFB1 |
